# Supplementary material for: Stability of Superhydrophobicity and Structure of PVDF Membranes Treated by Vacuum Oxygen Plasma and Organofluorosilanisation
Source: Membranes (Basel). 2023 Mar 9;13(3):314. doi: 10.3390/membranes13030314 (PMC10054235; doi:10.3390/membranes13030314)
Supplement: Supplementary file 1 [file membranes-13-00314-s001.zip › membranes-2222111-supplementary.pdf]

Supplementary Material for

Stability of Superhydrophobicity and Structure of PVDF Membranes Treated by Vacuum Oxygen Plasma and Organofluorosilanisation

Ramón Jiménez-Robles [1], Marta Izquierdo [1,*], Vicente Martínez-Soria [1], Laura Martí [2], Alicia Monleón [2,3] and José David Badia [1,*]

[1] Research Group in Materials Technology and Sustainability (MATS), Department of Chemical Engineering, School of Engineering, University of Valencia, Avda. Universitat s/n, 46100 Burjassot, Spain
[2] Decarbonisation Department, Plastic Technology Institute (AIMPLAS), C/Gustave Eiffel 4, 46980 Paterna, Spain
[3] Department of Organic Chemistry, School of Chemistry, University of Valencia, Dr Moliner 50, 46100 Burjassot, Spain
* Correspondence: marta.izquierdo-sanchis@uv.es (M.I.); jose.badia@uv.es (J.D.B.); Tel: +34-96-35-43737 (M.I.); +34-96-35-44319 (J.D.B.)

S1. Experimental system for the long-term stability tests

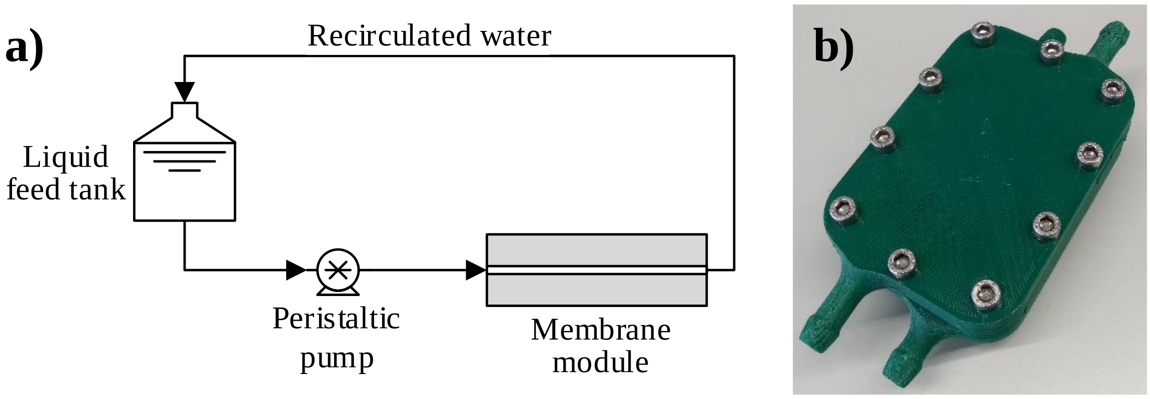

Figure S1. a) Scheme of the experimental system for the long-term stability tests with a flat-sheet membrane module using distillate water and b) image of the 3-D printed flat-sheet module used in the tests.

S2. Infrared spectra of the pristine PVDF and modified membranes with oxygen plasma activation and functionalisation

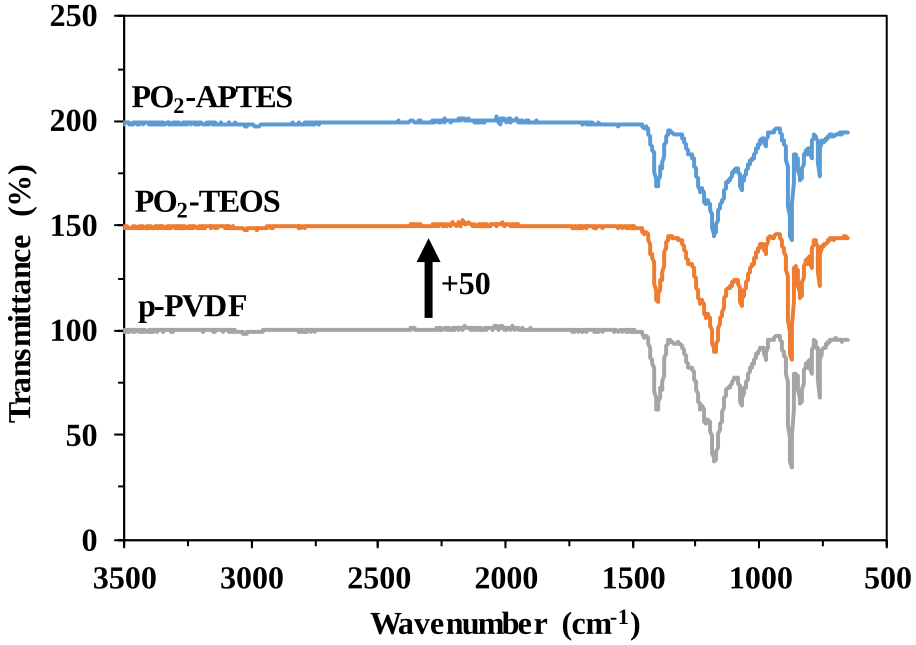

Figure S2. Infrared spectra of the pristine PVDF (p-PVDF) and modified membranes with O_2 plasma activation at 15 W during 15 min and functionalised with FAS/TEOS (PO_2-TEOS) and FAS/APTES (PO_2-APTES) obtained from the Fourier transform-infrared (FTIR) spectrometer in the attenuated total reflectance (ATR) mode.

S3. FESEM images at low magnification of the cross section of the pristine, coated and plasma treated PVDF membranes before the stability tests

FESEM images of the whole cross section of the pristine, coated and plasma treated PVDF membranes before the long-term stability test are shown in Figure S3 at a magnification of 500 – 600. FESEM images at a higher magnification of 2500 and focused on the surface are shown in the main paper (Figure 7).

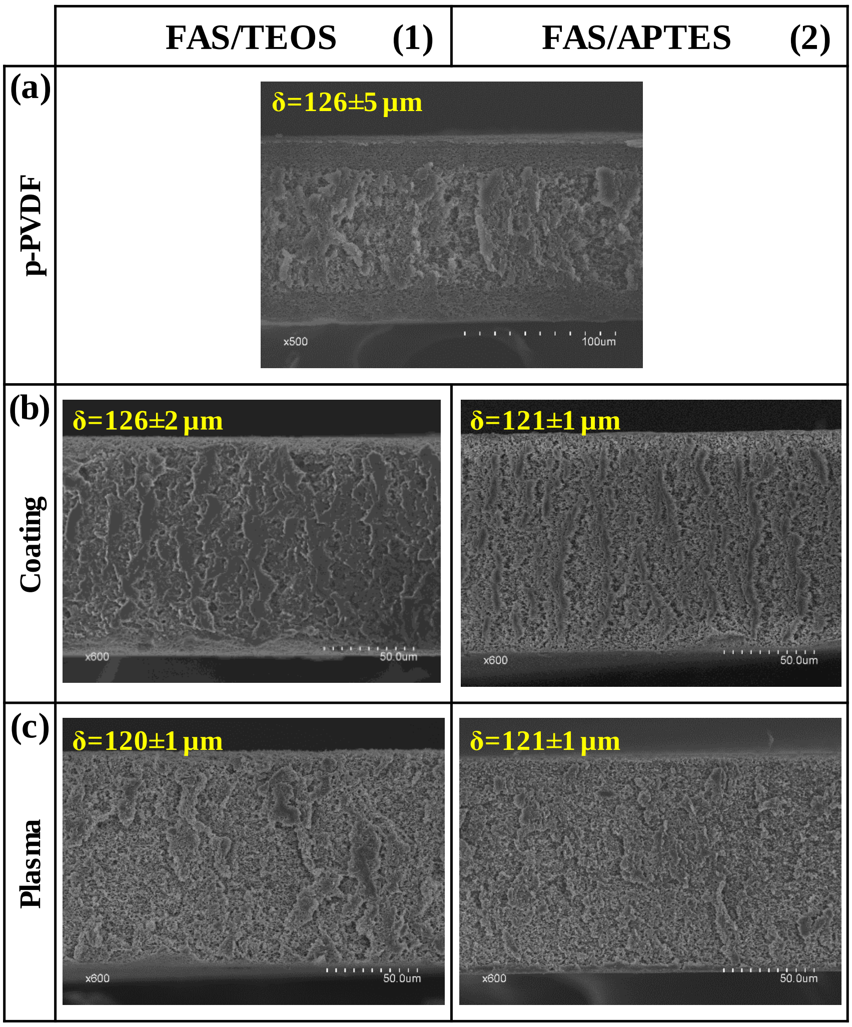

Figure S3. FESEM images of the cross section of the (a) pristine PVDF (p-PVDF), (b) coated PVDF membranes with (1) FAS/TEOS and (2) FAS/APTES and (c) modified membranes with O_2 plasma activation at 15 W during 15 min and functionalised with FAS/TEOS and FAS/APTES.
